# The role of micro RNAs (miRNAs) in the regulation of *Drosophila melanogaster*’s innate immunity

**DOI:** 10.1080/19336934.2022.2149204

**Published:** 2022-11-22

**Authors:** Max Yang Lu, Stanislava Chtarbanova

**Affiliations:** a Department of Biological Sciences, the University of Alabama, Tuscaloosa, AL, USA; b Center for Convergent Bioscience & Medicine, University of Alabama, Tuscaloosa, AL, USA; c Alabama Life Research Institute, University of Alabama, Tuscaloosa, AL, USA

**Keywords:** Micro RNAs (miRNAs), *drosophila melanogaster*, innate immunity, toll pathway, IMD pathway

## Abstract

MicroRNAs (miRNAs) are a class of small non-coding RNAs ~19–22 nt long which post-transcriptionally regulate gene expression. Their ability to exhibit dynamic expression patterns coupled with their wide variety of targets allows miRNAs to regulate many processes, including the innate immune response of *Drosophila melanogaster*. Recent studies have identified miRNAs in *Drosophila* which are differentially expressed during infection with different pathogens as well as miRNAs that may affect immune signalling when differentially expressed. This review provides an overview of miRNAswhich have been identified to play a role in the immune response of *Drosophila* through targeting of the Toll and IMD signalling pathways and other immune processes. It will also explore the role of miRNAs in fine-tuning the immune response in *Drosophila* and highlight current gaps in knowledge regarding the role of miRNAs in immunity and areas for further research.

## Introduction

Innate immunity acts as the first line of host defence against pathogenic microorganisms. Proper regulation of associated innate immune responses is vital, as sufficiently potent activation is necessary to protect against invading pathogens, while it is also important to prevent overactive immune responses that could result in tissue damage [1].

**Figure 1.**
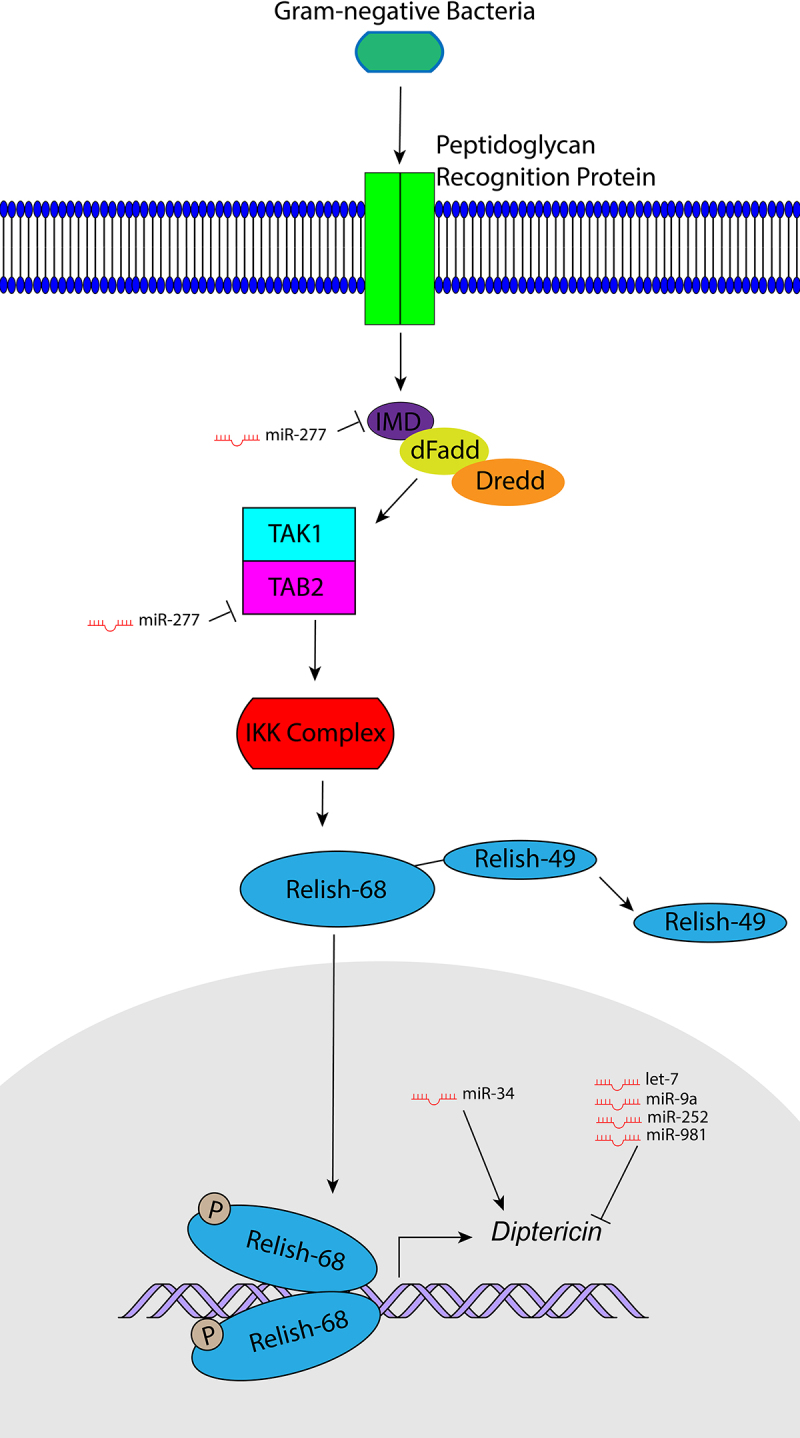
An overview of the IMD pathway and its regulation by miRNAs. Recognition of Gram-negative bacteria results in the formation of a complex with Imd, dFadd, and Dredd activating the Tab2/Tak1 complex. Subsequent activation of the IKK complex leads to phosphorylation of and cleavage of Rel which allows Rel-68 to translocate to the nucleus and activate transcription of *Dpt. miR-277* represses *imd* and *Tab2; miR-34* induces *Dpt* expression; *let-7, miR-9a, miR-252*, and *miR-981* repress *Dpt* expression.

**Figure 2.**
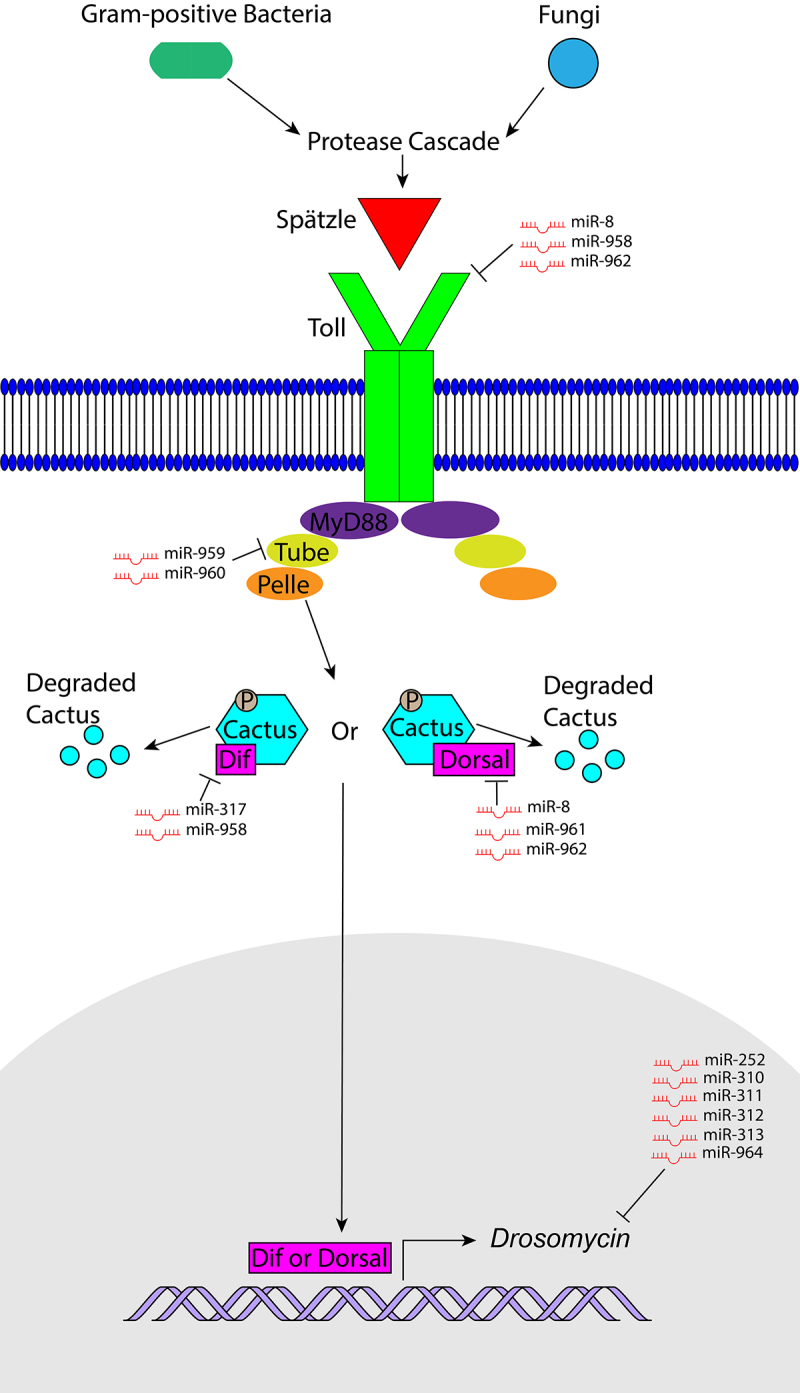
An overview of the Toll pathway and its regulation by miRNAs. Fungal and Gram-positive bacterial infections trigger a protease cascade leading to cleavage of pro-Spatzle to the ligand Spatzle which binds the Toll receptor. The formation of a MyD88-Tube-Pelle complex bound to the Toll receptor leads to phosphorylation and degradation of Cactus, allowing Dorsal and Dif to translocate to the nucleus and activate transcription of *Drs. miR-8, miR-958*, and *miR-962* repress *Toll; miR-959* and *miR-960* repress *Tube; miR-317* and *miR-958* repress *Dif; miR-8, miR-961* and *miR-962* repress *Dorsal; miR-252, miR-310-313* and *miR-964* repress *Drs.*

Lacking an adaptive immune system, the effects of innate immunity can be easily isolated in the fruit fly *Drosophila melanogaster*, making it an attractive model organism to study innate immunity. The two *Drosophila* Nuclear Factor kappa B (NF-κB) pathways Toll and Immune deficiency (IMD) (Figures 1 and 2) are evolutionarily conserved and homologous to the mammalian Toll-like receptor (TLR)/Interleukin-1 Receptor (IL-1 R) and Tumour Necrosis Factor Receptor (TNFR) pathways, respectively [2,3]. In *Drosophila*, these pathways lead to the secretion of a variety of antimicrobial peptides (AMPs) in response to bacterial and fungal infections and have also been implicated in antiviral immunity [4,5]. Expression of the antifungal peptide Drosomycin (Drs) is primarily regulated by the Toll pathway, while expression of the peptide Diptericin (Dpt), which defends against Gram-negative bacteria, is primarily regulated by the IMD pathway [6], allowing expression of the genes encoding for these peptides to be used as measures of Toll and IMD signalling activation respectively. While largely independent, cross-regulation does occur between the Toll and IMD pathways [7]. Among AMPs whose expression depends on both pathways we find AttacinA (AttA) and Cecropin that act against Gram-negative bacteria, Defensin that acts against Gram-positive bacteria, and Metchnikowin (Mtk) which is an antifungal peptide [7,8].

Accumulating evidence over the past decade suggests the ability of microRNAs (miRNAs), to regulate innate immunity in *Drosophila melanogaster*. miRNAs are a group of endogenous, small non-coding RNAs ~19–22 nt long which post-transcriptionally repress gene expression through complementary base pairing between the seed region (nt 2–7) of the miRNA and the 3’ UTR of target mRNA [9]. Such repression occurs either via a mechanism that directly degrades the mRNA or an alternate mechanism that simply induces its decay [10]. Due to the short length of the seed region, each gene is capable of being regulated by multiple miRNAs, and each miRNA is capable of regulating a diverse set of hundreds of transcripts, with miRNAs being estimated to target around 30% of all *Drosophila* genes [11,12].

miRNAs are implicated in many biological processes, including innate immunity. Their biogenesis begins in the nucleus with transcription of primary miRNAs (pri-miRNAs) by RNA Polymerase II [13,14]. Pri-miRNAs are subsequently processed by a complex involving the nuclear RNase III Drosha and its co-factor Pasha to produce a ~ 60–70 nt stem-loop structure known as a precursor miRNA (pre-miRNA). The pre-miRNA is then exported to the cytoplasm upon formation of a complex consisting of the pre-miRNA, the Exportin 5 protein, and its co-factor Ran-GTP [15–17]. Further processing by the RNAse III enzyme Dicer-1 into a double-stranded duplex results in two potentially functional miRNAs [18] termed the 3p and 5p species based on whether they are derived from the 3’ or 5’ arm respectively [19]. Subsequent loading into a member of the Argonaute (AGO) family of proteins, primarily AGO1 but also AGO2 [20], forms an RNA-induced silencing complex (RISC) which then acts to silence its targets [21,22]. *Drosophila* with disrupted miRNA biogenesis pathways are more vulnerable to infection with *Candida albicans* (*C. albicans*) and experience differential expression of AMPs when infected with *C. albicans* and *Escherichia coli* (*E. coli*) [23,24]. This indicates that miRNA processing is an important mechanism in protecting flies against infections and suggests a role in innate immunity of these regulatory RNAs.

*Drosophila* represent an excellent model for investigating miRNA-mediated regulation of innate immunity given their low cost of culture, short lifespan and developmental time, large number of offspring, and a small, easily manipulated genome. The binary Gal4/UAS expression system can be used in *Drosophila* for tissue and time-specific overexpression or knockdown of genes, including miRNAs [25,26]. There also exist a wide variety of tools for evaluating the effects of miRNA deficiency *in vivo*, including miRNA deletion mutants and miRNA-sponge mutants that express an oligonucleotide sponge construct that inhibits miRNAs via complementary binding [27,28]. Several miRNA target prediction algorithms have also been developed, including TargetScan, miRanda, PicTar, RNA22, and PITA [28]. Although these algorithms may sometimes provide false-positive results regarding miRNA–gene regulatory relationships, the application of various screening strategies can aid in the identification of relevant miRNA–gene interactions [29]. *In vitro* cell culture-based techniques such as luciferase reporter assays can also be applied. These reporters consist of fusing the predicted miRNA target sequence of the gene of interest (most often located in the 3’-untranslated region (3’-UTR) of the corresponding transcript) with the *luciferase* gene [30]. miRBase (www.miRbase.org), a database containing 258 miRNAs of *Drosophila melanogaster* in the latest version (v22.1) also provides an abundance of information on miRNAs, including annotations of potential miRNA targets, sequences, references in literature, deep sequencing data, and genome coordinates. Several other technologies are also used for the detection and quantification of miRNAs, including transcriptional reporters, *in situ* hybridization, northern blotting, quantitative real-time PCR (qRT-PCR), microarrays, and small RNA-sequencing [31]. This review will focus on the role and mechanisms by which microRNAs regulate innate immunity in *Drosophila melanogaster*.

## Regulation of *Drosophila* Immune Deficiency (IMD) pathway by miRNAs

miRNAs are able to regulate innate immune responses mediated by the IMD pathway, which primarily responds to Gram-negative bacterial infections (Figure 1) [3]. IMD signalling is initiated when peptidoglycan receptor proteins recognize *meso*-diaminopimelic acid (DAP)-type peptidoglycan found in most Gram-negative bacteria [32–35], leading to formation of an intracellular complex involving the immune deficiency (Imd) protein, the adaptor protein dFadd and the caspase Dredd [3,36–39]. The formation of this complex results in the activation of the TGF-β activated kinase 1 (Tak1)/Tak1-associated binding protein 2 (Tab2) complex [3,40,41], which works to activate the IKK kinase complex composed of the proteins Kenny (Key) and Ird5 [41–44]. The active IKK complex then phosphorylates the NF-kB transcription factor Relish (Rel) resulting in its cleavage into Rel-68 and Rel-49 subunits [45], allowing Rel-68 to translocate to the nucleus and promote transcription of its target genes of the IMD pathway including AMPs [46].

*In silico* analysis indicated the ability of the miRNA *let-7* to directly repress the IMD pathway AMP *Dpt*. This was validated using a luciferase reporter assay in cultured S2 cells [47], a cell line derived from embryonic macrophage-like cells [48]. In S2 cells, the steroid hormone 20-hydroxyecdysone (ecdysone) is capable of inducing *let-7* signalling through an Ecdysone receptor (EcR) and Broad Complex (Br) dependent pathway [47,49,50]. The immediacy of such a transcriptional response is highly dependent on prior priming and exposure to ecdysone [47], thus explaining why in experiments without priming, ecdysone signalling has a significantly delayed effect on *let-7* transcription [49,51]. Surprisingly, in S2 cells, while ecdysone induces *let-7* expression, it also induces expression of *Dpt*, a target that *let-7* represses. Indeed, ecdysone has been previously shown to enhance AMP gene expression, including *Dpt* expression, following pathogen challenge by regulating the IMD pathway in both cultured cells and *in vivo* [52–56]. This led Garbuzov and Tatar, 2010 to suggest a model in which ecdysone induces *Dpt* expression but *let-7* acts to prevent *Dpt* hyperactivation [47].

A separate report confirmed the effect EcR and Br have on mediating ecdysone signalling and the ability of such signalling to repress transcription of *miR-34* [24]. EcR, Br, and the transcription factors Serpent (Srp), Twist (Twi), and Apterous (Ap) were shown to mediate ecdysone repression of *miR-34*. A number of cis-regulatory elements near the *miR-34* locus, one of which is a Br target, also appear to be involved. Ecdysone represses *miR-34* transcription, but *miR-34* also represses *br* of the ecdysone signalling pathway, suggesting a positive feedback loop on *miR-34* transcription constituted by ecdysone.

*miR-34* acts as a positive regulator of IMD signalling. *In vivo*, ubiquitous *miR-34* overexpression results in increased levels of *Dpt* transcripts in both the presence or absence of *E. coli* infection [24]. *In vitro*, in comparison to controls, ecdysone-treated S2 cells overexpressing *miR-34* exhibit upregulated expression of several components of the IMD signalling pathway in both the presence and absence of peptidoglycan treatment. Among these, we find the AMPs *Dpt, Cecropin A1* (*CecA1), AttA, Defensin (Def)* and *Mtk*, and the *poor Imd response upon knock-in* (*pirk*) mRNA. Silencing of IMD pathway components in *miR-34* overexpressing S2 cells treated with ecdysone is associated with significantly decreased *Dpt* levels, indicating that *miR-34*-mediated modulation of *Dpt* expression occurs at least partially through the IMD pathway. Of note is the fact that ecdysone-treated S2 cells overexpressing *miR-34* require peptidoglycan-recognition protein LC (PGRP-LC) to exert their immune-stimulating effects, even in the absence of peptidoglycan treatment. Due to the manner in which ecdysone is able to trigger PGRP-LC expression [53], it is possible that *miR-34* mediates PGRP-LC expression in ecdysone signalling. Experiments involving *miR-34* ubiquitously overexpressing and *miR-34* knockout flies infected with pathogens including Gram-negative *Erwinia carotovora carotovora* strain 15 (*Ecc15), Enterobacter cloacae* (*E. cloacae*) and *E. coli* revealed that *miR-34* enhances immune responses by promoting increased AMP expression and pathogen resistance independently of phagocytic activity [24]. Additional *in silico, in vitro*, and *in vivo* analysis showed that the immune regulatory effects of *miR-34* are in-part due to direct repression of the genes *discs large 1 (dlg1), Su(Z)12, CG8468, murashka (mura), Ecdysone-induced protein 74EF (Eip74EF)* and *Ecdysone-induced protein 75b (Eip75B)*.

The diverse targets of *miR-34* signalling complicate our understanding of the exact mechanisms by which they regulate immunity. While the *miR-34* targets *Eip75B, dlg1, CG8468, mura* and *Su(Z)12* act as negative regulators of IMD signalling [24,40], the *miR-34* target *Eip74EF* acts as a positive regulator of IMD signalling [53]. It is possible that *miR-34* targets different genes during various stages of infection; however, more work is necessary to evaluate the temporal expression of its target genes during infection. Conflicting reports exist regarding changes in *miR-34* expression in response to Gram-negative *E. coli* infection. One report found no changes in expression from 0 to 120 hours post infection [24], and another study identified decreased expression at 24 hours post infection [57], making the role of *miR-34* in defending against acute *E. coli* infection unclear.

*miR-34* belongs to a cluster in the genome that also includes *miR-277*, a negative regulator of the IMD pathway that is repressible via ecdysone signalling [24,58]. However, the relationship between ecdysone and IMD signalling regulated via *miR-277* has not been well explored. One report identified that following *E. coli* infection, the *Drosophila* transcription factor Myc (dMyc) negatively regulates *Dpt* expression and IMD signalling through a pathway mediated by *miR-277* [58]. A chromatin immunoprecipitation (ChIP)-qPCR assay showed that dMyc activates *miR-277* expression via direct binding to its promoter, and a luciferase reporter assay in S2 cells demonstrated that *miR-277* directly targets *imd* and the *Tab2-Ra/Rb* isoforms whose products positively regulate IMD signalling [39,40]. *In vivo*, ubiquitous *miR-277* overexpression downregulates both *imd* and *Tab2* expression and can be rescued by the ubiquitous co-overexpression of a *miR-277* sponge construct [58]. In the presence of *E. coli* infection, the downregulated *Dpt* expression in *miR-277* overexpressing flies can also be rescued by co-overexpression of the *miR-277* sponge construct. Phenotypically similar to the *miR-277* overexpressing line is a *dMyc* ubiquitously overexpressing line that displayes decreased expression of *Dpt, imd*, and *Tab2* after *E. coli* infection. The expression of these genes is rescued in a *dMyc* and *miR-277* sponge ubiquitously co-overexpressing line [58]. Examination of temporal patterns of gene expression in *wild-type* flies infected with *E. coli* revealed that *dMyc* and *miR-277* exhibit opposite expression patterns from *imd* and *Tab2*, suggesting their ability to negatively regulate *imd* and *Tab2. dMyc* and *miR-277* display an initial drop in expression after infection but become upregulated in the later stages of infection. This suggests they may initially become downregulated to allow for an adequately potent immune response but become upregulated in the later stages of infection to prevent overactivation of the immune system and restore immune homoeostasis. However, despite dMyc’s role as a negative regulator of the IMD pathway, *dMyc* overexpressing flies show enhanced survival compared to controls in response to *E. cloacae* infection [58]. It is possible that dMyc may have other immunomodulatory roles independent of its effects on *miR-277* expression, which are responsible for such enhanced survival; however, further studies are needed to explore this possibility.

Investigation of the *miR-959-964* cluster, which undergoes circadian cycling, revealed that these miRNAs act as negative regulators of immune function [59]. *In vivo* experiments using *miR-959-962* knockout mutants (*miR-963* and *miR-964* were not knocked out) and ubiquitously overexpressing *miR-959-964* flies, showed that the cluster negatively affects resistance to pathogenic Gram-negative *Pseudomonas aeruginosa (P. aeruginosa)* infection. Indeed, *miR-959-962* knockout mutants infected with *P. aeruginosa* display varying circadian time-dependent survival, implicating cycling *miR-959-962* levels in the time-dependent regulation of immunity. The role of immunity for this miRNA cluster is supported by the fact that the fat body, a major organ in immune function [60], is a prominent site of *miR-959-964* cluster expression [59].

*miR-9a* and *miR-981*, two miRNAs upregulated in response to *E. coli* infection, also act as negative regulators of the IMD pathway [57]. Compared to controls, fly lines ubiquitously overexpressing *miR-9a* and *miR-981,* respectively, exhibitdecreased levels of *Dpt* in response to *E. coli* infection. In these lines, *the Dpt* expression is rescued by ubiquitous co-overexpression of each miRNA’s sponge. *In silico* predictions and luciferase reporter assay in S2 cells validated the ability of *miR-9a* and *miR-981* to directly target *Dpt* transcripts. *miR-9a* overexpressing flies also exhibitincreased *AttA* expression, while *miR-981* overexpressing flies exhibitincreased *CecA1* and *AttA* expression. Such upregulation of these AMPs was speculated to compensate for the reduced *Dpt* levels. The fact that *in vivo*, both *miR-9a* and *miR-981* which act as negative regulators of the IMD pathway are not differentially expressed until their upregulation during the end phase of immune response induction (24 hours post infection), suggests that they maintain immune homoeostasis and prevent immune overactivation.

## Regulation of *Drosophila* Toll pathway by miRNAs

In *Drosophila*, miRNAs also regulate the Toll pathway, which is primarily stimulated by fungal and Gram-positive bacterial infections (Figure 2) [61]. Once the pathway is triggered, extracellular protease cascades lead to cleavage of pro-Spatzle to generate the ligand Spatzle which subsequently binds a Toll receptor [62–64]. In the cytoplasm, the adaptor protein MyD88 binds the activated Toll receptor, and recruits another adaptor: Tube, and the kinase Pelle to form the MyD88-Tube-Pelle protein complex [65]. Downstream of this complex is the inhibitor of NF-κB (IκB) factor Cactus and NF-κB transcription factors Dorsal and Dorsal-related immunity factor (Dif), which in resting conditions are bound to Cactus in the cytoplasm and prevented from transcribing their target genes [66,67]. The formation of the MyD88-Tube-Pelle protein complex results in phosphorylation and degradation of Cactus [68], allowing Dorsal and Dif to translocate to the nucleus and activate transcription of various genes such as AMPs [61,66,67,69,70].

Using a collection of miRNA mutants, a genetic screen identified miRNAs involved in the *Drosophila* response to *C. albicans* infection and Toll signalling. The authors found significantly increased *Drs* expression in *miR-277-34* mutants. These flies display increased *Drs* expression after injection with PBS, which is rescued by compromising the Toll pathway component Dif. To separate the effects of *miR-277* from *miR-34*, expression levels of *Drs* were examined in an immune tissue-specific *miR-277* sponge overexpression line. These flies display upregulated *Drs* levels , in line with the *in silico* prediction of *miR-277’*s ability to directly target *Drs* [23]. The opposing effects on *Drs* levels found in *miR-277-34* mutants and *miR-277* sponge overexpressing flies suggest opposite effects of *miR-277* and *miR-34* on Toll signalling and that *miR-34* stimulates innate immune responses as observed for the IMD pathway [24]. However, the role of *miR-34* on Toll signalling remains unsettled as Xiong et al., 2016 found that following Gram-positive *Micrococcus luteus* (*M. luteus*) infection, flies ubiquitously overexpressing *miR-34* exhibit decreased *Drs*, suggesting that *miR-34* downregulates the Toll pathway instead.

The *miR-310-313* cluster is another miRNA cluster that, when knocked out, results in an increased susceptibility to *C. albicans infection* [23]. Interestingly, a separate report found that each member of the *miR-310-313* cluster is capable of negatively regulating Toll signalling through direct targeting of *Drs* [71]. The *in silico* predictions of such targeting were validated using luciferase reporter assay in S2 cells, and *in vivo* using fly lines that either overexpressed or were deficient in a singular member of the miRNA cluster. Overexpression of the entire cluster represses *Drs* more strongly than overexpression of any singular miRNA within the cluster, suggesting that these miRNAs work together synergistically to repress *Drs*. Examination of temporal patterns of *Drs* and miRNA expression in *M. luteus* infected flies revealed that *Drs* peaked at 12 h post-infection and showed upregulation of *miR-310* at 48 h, upregulation of *miR-311* at 24 and 48 h, upregulation of *miR-312* at 24 h, and upregulation of *miR-313* at 12 h post-infection. The upregulation late in the course of infection suggests a role in preventing immune overactivation of this cluster of miRNAs.

The *miR-959-964* cluster, which plays a role in defence against Gram-negative bacteria [59], also possessess roles in Toll signalling. One study found that ubiquitous *in vivo* overexpression of *miR-963* and *miR-964* results in reduced *Drs* expression during *M. luteus* infection [71]. Another report showed decreased survival and decreased *Drs* expression for flies ubiquitously overexpressing *miR-959-962* in response to Gram-positive *Enterococcus faecalis* (*E. faecalis*) infection and increased survival and *Drs* expression in *miR-959-962* knockout flies [72].

An individual member of the *miR-959-964* cluster, *miR-964*, was identified as a negative regulator of Toll pathway signalling. Ubiquitous *in vivo* overexpression of *miR-964* results in increased susceptibility to *E. faecalis* infection and reduced *Drs* expression during Gram-positive *M. luteus* infection, and can be rescued by ubiquitous co-overexpression of a *miR-964* sponge [73,74]. *In silico* prediction of *miR-964* targets and a confirmation study using a luciferase reporter assay in S2 cells showed that *miR-964* directly targets *Drs. miR-964* is a miRNA that is differentially expressed during infection, however, unclear patterns of expression following infection make it difficult to discern its specific role. qRT-PCR-based gene expression analysis determined *miR-964* to be upregulated in response to *M. luteus* infection at 24 h, 48 h, and 72 h post-infection [73]. In other reports, at 24 h post-infection, *miR-964-5p* is downregulated while expression of *miR-964-3p* is not differentially changed to statistically significant levels [71,75]. It is possible that circadian cycling of the cluster may impact measurements of expression for miRNAs within the cluster [59].

Individual miRNAs within the *miR-959-962* cluster can also act as negative regulators of Toll signalling [72]. Ubiquitous *in vivo* overexpression of each individual miRNAs results in heightened susceptibility to *M. luteus* infection and decreased *Drs* expression. *In silico* prediction and validation using a luciferase reporter assay in S2 cells, and ubiquitous miRNA overexpression *in vivo* show that *miR-959* and *miR-960* directly repress *tube, miR-961* and *miR-962* repress *dorsal*, and *miR-962* represses *Toll*, all three genes encoding components of the Toll signalling pathway. The dynamic expression of members of this miRNA family was also examined in response to *M. luteus* infection. Members of the cluster exhibit increased expression only 24 h and 48 h post-infection in the late infection stages. Given the negative regulatory role of these miRNAs, this suggests the role of members of this cluster in re-establishing immune homoeostasis and preventing immune hyperactivation. It was speculated that because miRNAs from the *miR-959-962* family are transcribed from the same intron and exhibit similar temporal patterns of expression [59] yet different temporal patterns of repression, the individual miRNAs may have varied half-lives to further fine-tune regulation of Toll signalling. Ubiquitous overexpression of all members of the *miR-959-962* family *in vivo* has a greater effect on *Drs* repression than overexpression of a singular miRNA of the family, suggesting that these miRNAs work together synergistically to exert their regulatory functions.

*miR-317* has also been implicated in negative regulation of Toll signalling during infection [76]. The use of *miR-317* ubiquitously overexpressing flies, *miR-317* knockout, and a *miR-317* and *miR-317* sponge co-overexpressing line suggests that *miR-317* negatively regulates *Drs* during *M. luteus* infection through targeting of the Toll signalling component *Dif* [66,71,74]. Specifically, *miR-317* targets the *Dif-RC* isoform but not the *Dif-RA/RB/RD* isoforms, which have different 3’ UTRs and exons [76]. The ability of *miR-317* to target the *Dif-RC* isoform selectively and directly was subsequently confirmed via *in silico* predictions and a luciferase reporter assay in S2 cells. *miR-317* expression impacts the survival of *E. faecalis* infection with *miR-317* ubiquitously overexpressing flies and *miR-317* knockout mutants exhibiting decreased and increased survival, respectively. However, reports showing inconsistent patterns of *miR-317* expression in *wild-type* flies make it difficult to evaluate the complete role of *miR-317* in immunity. One report employing qRT-PCR found *miR-317* to be upregulated at 24 h post-infection with *M. luteus* [76], which contradicts previous small RNA-seq data demonstrating downregulation of both 3p and 5p species of *miR-317* 24 h after *M. luteus* infection [71,75].

*miR-958* is another miRNA known to target specific isoforms of components of the Toll signalling pathway [74]. *miR-958* ubiquitously overexpressing flies display lower levels of *Drs* during *M. luteus* infection and increased susceptibility to *E. faecalis* infection, while *miR-958* null mutants show higher levels of *Drs* during *M. luteus* infection [71,74]. *In silico* predictions suggest that *miR-958* targets all *Toll* and *Dif* isoforms except *Dif-RC,* which was verified via a luciferase reporter assay in S2 cells. *In vivo,* the ubiquitous overexpression of *miR-958* revealed that the downregulation of *Toll, Dif-RA* and *Drs* expression in these flies could be rescued by the ubiquitous co-overexpression of *miR-958* sponge. Given that *Dif-RA, Dif-RB*, and *Dif-RD* have identical 3’ UTR sequences, it is likely that *miR-958* can repress all of these isoforms. Patterns of expression during infection of *miR-958* are unclear, making it difficult to fully discern its role. In *wild-type* flies infected with *M. luteus*, the upregulation of *miR-958* was shown to occurr late during the course of infection at 12 h, 24 h and 48 h post infection [74]. In another study, it was found that at 12 h post infection neither *miR-958-3p* nor *miR-958-5p* were differentially regulated and at 24 h, *miR-958-3p* was downregulated [71,75].

*miR-8* is also implicated in negative regulation of innate immune pathways, even in the absence of infection [77,78]. In the absence of septic injury, *miR-8* null flies exhibit increased basal levels of *Drs* and *Dpt* expression as well as increased frequency of spontaneous melanization [77], a sign of overactive immune pathways [79]. In the absence of infection, the *miR-8* null phenotype is detrimental to survival, with just 12% of the flies surviving to adulthood [78]. This phenotype of the *miR-8* null mutants could be partially reversed by fat body-specific knockdown of *dorsal*, as well asubiquitous knockdown of *spatzle* and *u-shaped* (*ush*) respectively, suggesting a negative regulatory role for *miR-8* in Toll signalling and repressing *ush* to maintain immune homoeostasis and survival. *In silico* predictions and validation via a luciferase reporter assay in S2 cells showed that *miR-8* directly targets *Toll* and *dorsal* transcripts [78]. *miR-8* represses *ush*, a known binding partner of GATA transcription factors involved in AMP gene transcription, which also acts as a repressor of Phosphoinositide 3-Kinase (*PI3K*), a component of the insulin signalling pathway [80–87]. Fly lines overexpressing *dorsal* and *ush* in the fat body (the lack of a *Toll* overexpressing line prevented its testing) showed that these two factors mediate *miR-8* regulation of *Drs* expression [77,78]. However, neither *Drs* nor *Dpt* were elevated in two other mutant lines with ubiquitous and fat body-specific inhibition of PI3K [77], suggesting the immunoinhibitory role of *miR-8* is not mediated through PI3K. *Drs* upregulation in *miR-8* null larvae is largely dependent on fat body *miR-8* levels but not other important immune tissues such as the epidermis and the gut [77]. *miR-8* nulls in which *miR-8* expression is restored solely in the fat body exhibit rescued *Drs* levels throughout the body of larvae as well as rescued *Drs* and *Dpt* levels in adults and reduced prevalence of melanization.

## Roles of miRNAs in other aspects of *Drosophila* immunity

In addition to Toll and IMD pathway-mediated regulation of immunity, miRNAs are implicated in regulation of many other genes with roles in innate immunity in *Drosophila. miR-956-3p* acts as a negative regulator of immunity via repression of *Sterile alpha and Armadillo motif* (*Sarm* or *Ect4*) [88]. Sarm is a Toll/Interleukin-1 Receptor domain-containing protein that contributes to limit IMD pathway activation in *Drosophila* respiratory epithelial cells during bacterial infection [89]. Mutants carrying a deletion of *miR-956*, a miRNA whose 3p species is significantly downregulated 3 days post infection with the RNA virus *Drosophila* C Virus (DCV), exhibit enhanced survival and decreased virus load after DCV infection [88]. Although the deletion of *miR-956* removes both the 3p and 5p species, given the low levels of *miR-956-5p*, such phenotypical changes are likely due to the more abundant *miR-956-3p* [90,91]. *In silico* analysis and assays using S2 cells treated with synthetic *miR-956-3p* mimics show that *miR-956-3p* represses *Sarm* [88]. Both *wild-type* and *miR-956* mutant flies upregulate *Sarm* expression after DCV infection; however, *miR-956* mutants induce *Sarm* expression to a lesser extent than *wild-type* flies. This suggests that *Sarm* induction during DCV infection is *miR-956* dependent. Further experiments demonstrating decreased survival and increased virus load following DCV infection in *Sarm* mutants suggest that the decreased *miR-956* levels exhibited by *wild-type* flies in response to DCV infection derepress *Sarm* and promote innate immunity.

*miR-8-5p*, which regulates Toll signalling [77,78], also promotes viral resistance through repression of *Jun-related antigen* (*Jra* or *dJun*) [92]. *In silico* prediction and a *Spodoptera frugiperda* (Sf9) insect cell culture-based reporter assay using the *GFP* gene cloned upstream of the *Jra* 3’UTR sequence, confirmed that *miR-8-5p* represses *Jra*. In concordance with these results, *Jra* is upregulated during DCV infection, aligning with the observed *in vivo* and *in vitro* downregulation of *miR-8-5p* during DCV infection [88,92]. *In vitro* knockdown of *miR-8-5p* and *Jra* in S2 cells demonstrates that these two factors promote decreased and increased viral titres, respectively. The role of *Jra* in promoting viral accumulation and modulating immunity is not yet well characterized, but it has been speculated that Jra may induce host factors involved in viral infection or regulate the apoptosis pathway.

While both *miR-8-5p* and *miR-956-3p* are downregulated during DCV infection [88,92], it is not entirely clear whether such decreases are part of the host immune response or caused by the general degradation of RNA during DCV infection [93]. Another miRNA, *miR-305-3p*, is strongly induced in S2 cells upon infection with the DNA virus invertebrate iridescent virus 6 (IIV6) with a proposed role in virus accumulation [93]. Possible targets of this miRNA include *PI3K* and the *Drosophila* homolog of *p53* which are often used by viruses in replication [94–97].

miRNAs also play an immune role through maintaining gut barrier function. Loss of function of the *miR-34* target *dlg1* [24], a mediator of *miR-34* repression of IMD signalling, is implicated in gut barrier dysfunction [98,99]. Such dysfunction from the loss of *dlg1* expression may result in increased leakage of microbes and subsequent upregulation of AMPs [24]. *miR-263a* also plays an immune-relevant regulatory role in gut function [100]. *miR-263a* null flies display increased expression of IMD and Toll-related genes including *AttA, Dpt, Rel*, and *Drs* as well as increased bacterial loads in the midgut after oral infection with *P. aeruginosa. miR-263a* mutants also exhibit increased susceptibility to such infection [100], potentially a consequence of the reduced structural integrity of the peritrophic matrix, an insect midgut structure that has previously been shown to play a role in susceptibility to bacterial infection [101].

*miR-252-5p* works cooperatively with the Forkhead box O (FoxO) transcription factor to modulate innate immunity in the absence of infection through the gene *Dawdle* (*Daw*), which encodes a ligand of the TGF-β pathway [102]. The ability of FoxO to target and repress *Daw* has previously been established [103], and further *in silico* analysis and confirmation via a luciferase reporter assay in S2R+ cells, an isolate of the S2 cell line [104], show that *Daw* is a *miR-252-5p* target [102]. *In vivo* experiments have addressed the cooperative ability of *miR-252-5p* and *FoxO* in downregulating immune activity [102]. Flies with ubiquitous knockdown of either *miR-252* or *FoxO* alone display increased prevalence of melanization in the absence of injury, but the greatest prevalence of melanization is found in flies with ubiquitous knockdown of both *miR-252* and *FoxO* (double mutants). This is further supported by RNA-sequencing and gene expression analysis of these mutant lines. Single-cell sequencing of the double mutants also uncovered increased levels of haemocytes, a class of immune macrophage-like cells [105], further validating the upregulation of immune responses in these flies. It was suggested that *miR-252*-mediated downregulation of immune activity is mediated through *Daw* as the ubiquitous knockdown of *Daw* in addition to *miR-252* and *FoxO* rescues heightened immune activation found in the double mutants [102]. A line with both deletion of *Daw*’s *miR-252* binding site and ubiquitous *FoxO* knockdown phenocopies *miR-252, FoxO* double mutants, revealing that *miR-252* regulation of immune activity is mediated through Daw rather than other mRNA targets. Downstream of *miR-252* and FoxO, Daw modulates immune responses by activating the transcriptional factor Smad on X (Smox) which represses *Autophagy-related 8a* (*Atg8a*), a gene in the autophagy pathway [103]. Reduced levels of *Atg8a* found in *miR-252, FoxO* double mutants, in combination with knockdown of either *Key* or *Rel* which regulate IMD signalling downstream of *Atg8a* [42], suggest a pathway in which *miR-252* and FoxO repress *Daw*, leading to upregulation of Atg8a activity. Subsequent decreases of Key and Rel activity then downregulate IMD signalling to attenuate inflammaging, a term referring to the low-grade chronic inflammation accompanying ageing, which has deleterious effects on lifespan [106]. Supporting this pathway, uninfected flies carrying one mutant copy of either *Key* or *Rel* in *miR-252, FoxO* double homozygous mutant background exhibit increased lifespan, reduced prevalence of melanization, and rescued levels of various Toll and IMD AMPs including *Drs, Mtk,* and *Drosocin* [102]. Flies overexpressing *Atg8a, miR-252*, and *FoxO* have reduced induction of innate immunity genes and increased lifespan, further validating the ability of these genes to negatively regulate inflammaging and innate immunity.

Examination of differentially expressed genes potentially regulated by miRNAs during *M. luteus* infection also implicates miRNAs in regulating various processes involved in immunity, such as phagocytosis and immune cell proliferation [75], although such regulatory targets of miRNA require further validation.

## Conclusions and outlook

In this article, we reviewed the plethora of impacts various miRNAs have on innate immunity as well as the mechanisms by which their expression is regulated. There are still many other miRNAs, which have been predicted to target immunity-related genes [57], been identified as differentially expressed during infection [23,57,71,88] or have been shown to impact immune activity [23,24,57,58,71,107]. However, more research is needed to confirm such roles and characterize potential mechanisms of regulation.

In the current body of literature, many studies suggest a role for miRNAs in downregulating immune responses, often during the late stages of infection, which results in maintaining immune homoeostasis and avoiding immune hyperactivation. A regulatory network developed through identification of inverse correlations in expression between target genes and potential miRNA repressors at 3 h, 12 h, and 24 h post infection suggests that differential expression of miRNAs at 3 h and 12 h post infection with *M. luteus* promotes a more potent immune response, while at 48 h post infection, just one miRNA of the network is downregulated in comparison to 36 upregulated miRNAs. These many upregulated miRNAs with a wide range of targets involved in immune signalling suggests that the role of miRNAs is in preventing immune overactivation [75]. Examination of these dynamic patterns of expression during infection also reveals that far more miRNAs are differentially expressed at later stages of infection than during earlier stages [23,57,75], causing Wei et al., 2018 to speculate that in the early stages of infection, rapid changes in mRNA may occur to mount a potent immune response, while miRNAs act later to fine-tune such responses [75]. While many miRNAs exhibit such dynamic patterns of expression during infection [57,75], they also impact the basal levels of AMPs [77,78]. The maintenance of immune homoeostasis is crucial, given the well-established trade-offs between immunity and metabolism, reproduction, lifespan, and developmental time [1].

Important areas of future research include the impact of miRNAs on age, time, and sex-dependent differences in immunity. Recently, miRNAs have also been implicated in inflammaging [102]. Many miRNAs exhibit differential regulation with age [108–110], circadian time [59], and sex [110]; however, more work is necessary to examine how such differential regulation affects immunity. In addition, most existing studies have examined the role of miRNAs in modulating innate immunity to acute bacterial infection, leaving the role of miRNAs in regulating immune responses to persistent infection and in antiviral immunity as major areas for future research. *mir-956-3p* and *miR-8-5p* have been implicated in antiviral immunity and flies deficient in *Arsenic resistance protein 2* (*Ars2*) and the nuclear *cap binding complex* (*CBC*), components involved in miRNA silencing were found to be more susceptible to viral infection [88,92,111]. The Toll and IMD pathways also play a role in antiviral immunity [4,5,112], suggesting a possible role for miRNAs in defence against viruses through modulating these pathways. Reciprocally, viruses have also been shown to impact miRNA expression and activity [88,92,113], suggesting a complex interplay between these two elements that requires further study. Evaluation of the dependence of miRNA expression based on pathogen type will also yield insights about which pathways different miRNAs may act in. The use of *Drosophila melanogaster* to study such relationships will prove invaluable in yielding insights regarding miRNA mediated regulation of innate immunity.

## Data Availability

Data sharing is not applicable to this article as no new data were created or analysed in this study.
